# The Issue of Screening for Transfusion‐Transmitted Infections in Sub‐Saharan Africa Presents Several Challenges: A Review

**DOI:** 10.1155/ah/1373786

**Published:** 2025-10-28

**Authors:** F. Y. Djientcheu Deugoue, M. Azabji-Kenfack, C. Tayou Tagny, M. A. Kouamou Tchiekou, S. A. Menteng Tchuente, Veronique Deneys

**Affiliations:** ^1^ Faculty of Medicine and Biomedical Sciences, University of Yaounde I, Yaounde, Cameroon, uy1.uninet.cm; ^2^ Hematology and Blood Transfusion Department, Yaounde University Hospital Center, Yaounde, Cameroon; ^3^ Higher Institute of Health Sciences, University of Mountains, Bangangte, Cameroon; ^4^ Faculty of Medicine and Dental Medicine, Catholic University of Louvain, Brussels, Belgium, uclouvain.be; ^5^ Blood Bank, Saint-Luc University Clinics, Brussels, Belgium, saintluc.be

**Keywords:** Africa, blood transfusion, challenges, screening, TTI

## Abstract

Blood transfusion is a vital medical procedure with the potential to save lives; however, it can have serious consequences if not carried out under rigorous principles. Sub‐Saharan Africa (SSA) faces considerable challenges in ensuring the security of the transfusion process, particularly in the context of transfusion‐transmissible infections (TTIs). These difficulties are principally associated with the elevated prevalence of TTI in the region, including viral (e.g., hepatitis B, HIV and hepatitis C), bacterial (e.g., syphilis), and parasitic (e.g., malaria) diseases, among others. Also, the family replacement donation system, economic and technological limitations and an insufficient donor pool constitute substantial obstacles. Various solutions are currently being recommended, implemented, or applied on a variable scale, depending on the specific country and subregion, to address the challenges above. For instance, it would be judicious to implement measures to prevent infections, such as rigorous donor selection, quality testing for the primary TTI, and pathogen inactivation. This would necessitate deploying innovative and adapted communication strategies to establish a sufficient pool of regular, unpaid voluntary donors and high‐quality screening. Therefore, robust and coherent national or regional blood transfusion policies should support the previously outlined measures. These policies should include rigorous quality control and haemovigilance systems and reflect local epidemiological and economic realities.

## 1. Introduction

Blood transfusion is a therapeutic procedure that has proven to be an effective means of saving millions of lives. Nevertheless, the procedure’s safety remains a significant concern that requires further investigation [1, 2]. Indeed, the global blood collection reached 118 million units in 2018 [3]. Among the collection countries, a minimum of 10 countries could not screen for one or more of the key transfusion‐transmissible infections (TTIs) in all donated blood samples [3]. The transfusion of blood obtained and screened through low‐resource systems is associated with an elevated risk of TTI. Insufficient economic resources are evident for purchasing and maintaining supplies and equipment, training and quality monitoring [4]. The consequence of unsafe blood transfusions represents a significant challenge for low‐income countries, including those in sub‐Saharan Africa (SSA) [5]. Moreover, excluding donors based on potential risk will result in restricted blood availability, which could ultimately lead to an inability to meet patients’ needs. This, in turn, may contribute to the existing tension between blood safety and availability [6]. One of the principal factors affecting the safety of blood transfusions in Africa is the high prevalence of infections transmitted through transfusion, which can have a devastating impact and generate considerable public interest [6–9].

To promote transfusion safety, the World Health Organization (WHO) recommends that all blood donations be screened for the main TTI, which includes human immunodeficiency viruses (HIV), hepatitis B (HBV), hepatitis C (HCV) and syphilis [3]. Furthermore, to minimise the risk of TTI, the African Society for Blood Transfusion (AfSBT) recommends rigorous screening of all donated blood for major TTI using internationally approved, validated test kits. According to these recommendations, testing algorithms must ensure high sensitivity and specificity without compromising recipient safety, and only nonreactive units should be eligible for transfusion [10]. The AfSBT also recommends aligning screening with national guidelines and regional disease profiles, enforcing strict protocols for testing procedures and managing reactive or false‐reactive donors. Finally, these requirements should be included in a national blood policy and supported by a robust legal framework [10].

Significant efforts are being made by African countries to achieve this goal. Accordingly, an analysis of two reports from the global database on transfusion safety and the WHO blood supply indicates that screening for at least one of the key diseases has been implemented in the majority of African countries, with a significant increase from less than 60% of donations before the year 2000 to nearly 100% (ranging from 94% to 99.9% for different pathogens) during the second half of the 2010s [3, 11]. Despite these advances, the risk of contracting such an infection through blood transfusion in many regions across the continent remains significant. It is estimated that up to 500 people contract TTI every day following a transfusion of contaminated blood in Africa [12, 13]. This is partly due to the high prevalence of the diseases in the general population and the absence or lack of adherence to national blood donation screening strategies, which are often inadequate or lack sufficient resources [12, 14].

Despite the advancements achieved over time, the availability of safe blood for transfusion remains a significant challenge for these SSA countries. This can be attributed to several factors, including those previously mentioned and the sustained use of replacement donors [4, 13, 15]. Additionally, the organisations responsible for blood transfusion in these countries must address the dual challenge of maintaining adequate blood product reserves, which are both sufficient in quantity and of an appropriate quality, while ensuring the safety of the blood supply. In response to this challenge, which encompasses the entirety of the blood safety chain, local and international initiatives to augment the supply of safe blood are either being implemented or require implementation by blood collection organisations [13, 15, 16].

## 2. Epidemiology of TTIs

When the blood transfusion procedure is not conducted safely, it can result in various infections for the recipient, including viral, bacterial and protozoal infections [17]. The risk is amplified in the African continent, where infectious diseases with a high potential for blood transmission are prevalent (Table 1) [14, 15, 18]. Since TTIs represent nearly 70% of all deferrals to blood donation, they are a significant challenge in maintaining an adequate blood supply [17, 19].

**Table 1:** Median proportion of major transfusion‐transmitted infections in Africa [28, 29].

Infection	General population (% [range])	Blood donor (% [range])
HBV	7.5 [5.7–10.5]	2.8 [0.05–8.6]
HCV	0.8 [0.6–1.4]	0.6 [0.0–3.9]
HIV	3.6 [2.9–4.2]	0.9 [0.03–2.3]
Syphilis	1.7 [1.4–2.0]	0.9 [0.04–6.4]

Abbreviations: HBV, hepatitis B virus; HCV, hepatitis C virus; HIV, human immunodeficiency virus.

The prevalence of TTI in blood donations varies considerably between African countries and regions [8, 12, 19, 20]. The underlying causes of this situation may be attributed to the existing differences in the prevalence of these infections in the general population across different countries and the quality of screening tools, as opposed to the efficacy of preventive measures or the effectiveness of the blood donor selection programme [12, 20]. Concerning the regional disparities in the transmission of certain TTIs, Abdella et al. [5] demonstrated a declining prevalence of HIV, HBV and HCV infections in Ethiopia. The authors attributed this decline to the implementation of prevention and care programmes, improvements in the quality of screening kits and a rigorous donor selection strategy [5]. Conversely, Quintas et al. [8] demonstrated a higher prevalence of TTI than previous studies conducted in Angola [8]. Such discrepancies can also be attributed to socio‐cultural dissimilarities, variations in the risk profiles of different populations, and disparate endemicity levels of these infections across various African countries [5, 21]. Nonetheless, HBV remains the most prevalent TTI, at times accounting for over half of all cases. Furthermore, replacement donors constitute the majority of individuals with TTI in their donations [8, 22–24].

Over the aforementioned classic viruses, there are other potential transfusion‐transmissible agents, including the human T‐lymphotropic virus, the hepatitis E virus, the arboviruses (West Nile virus, Zika virus, dengue virus and human herpes virus 8), and others [25]. While the transmission of these viruses through transfusion has been demonstrated, they are infrequently sought in donations by African transfusion establishments. Similarly, despite their known regional endemicity, bacteria and parasites are not always detected in blood donations intended for transfusion in SSA [25].

Concerning malaria, the prevalence among blood donors remains considerable, exceeding 30% in countries such as Benin and Kenya or 70% in Malawi [13, 26]. Though there is a dearth of robust response measures at the continental level, even though malaria represents one of the most significant transfusion‐associated infections in the SSA region [13, 26]. Such prevalence levels will probably have substantial consequences, particularly for vulnerable patients or those with a compromised immune system, principally since the exclusion of malaria‐positive or high‐risk blood donors may not be a viable option due to the high demand for blood transfusions [4, 26]. To date, only a limited number of countries in the SSA region have implemented a systematic approach to screening blood donations for malaria [27]. It thus falls upon each African country to implement measures aimed at identifying groups at high risk of malaria infection and to incorporate this information into the criteria used for donor selection [26]. Nevertheless, lack of data exists regarding the efficacy of different screening methods in detecting low‐level parasitaemia in donors. These include microscopy, rapid diagnostic testing and molecular techniques. These considerations are of particular importance, given the financial implications, the need for prompt results and the expertise of laboratory personnel [26, 27]. Moreover, there is an inadequate implementation of the WHO guidelines on prophylactic antimalarial administration before transfusion, due to financial limitations and the possible development of drug resistance [27]. It is therefore imperative that each country establish post‐transfusion management protocols based on evidence of infection, through the use of microscopy, for example. The identification of risk factors may facilitate the refinement of donor selection criteria [4].

## 3. Blood Donor Recruitment

The recruitment of blood donors represents a significant challenge to the long‐term sustainability of the global transfusion chain. This is especially the case in SSA, where current blood donation systems cannot keep pace with the region’s expanding transfusion requirements, driven by the introduction of universal health coverage and enhanced access to healthcare [14]. These countries face many challenges, including a lack of resources that impedes their ability to effectively recruit and retain blood donors, thereby hindering the implementation of programmes and interventions in this area [14].

The SSA encounters challenges in establishing a pool of regular volunteers who donate blood to supply blood banks [13]. Many potential strategies could be considered to address this issue and enhance the current supply levels. This would entail formulating rigorous selection policies for blood donors at the national or regional level (including voluntary donations and exhaustive screening for TTI in donors) and implementing innovative programmes, such as mobile telephone‐based strategies, to recruit and, above all, retain blood donors, which should be voluntary and unremunerated [13]. In addition to maintaining the participation of occasional donors, alternative strategies may be employed to reinforce the donor pool through targeted interventions. It would therefore be possible to implement measures to dispel the fears associated with donating blood among women, who remain under‐represented among donors [9, 13]. Moreover, to maintain a consistent supply of donors, it would be advantageous to provide comprehensive nutritional advice and consider iron supplementation following each donation, given that a low haemoglobin level is one of the most common reasons for deferral. This may lead to the reduction of donor exclusion due to anaemia [4, 13, 24].

A significant number of countries employ the practice of replacement donations, which is contrary to the fundamental principle that all blood donations must be voluntary and unpaid, without any form of coercion or obligation on the part of the donor [3]. This presents a significant challenge in terms of ensuring the safety of transfusions. Indeed, it has been demonstrated that this category of donors was more at risk of transmitting TTI due to a higher prevalence of said infections within the group in question [8, 17, 23]. However, initiatives are underway to reverse this trend. Indeed, the WHO estimates that nearly half of African countries collect more than 80% of their blood from voluntary, unpaid donors [13, 29]. Nevertheless, the figures demonstrate notable variability, ranging from 26.4% in the Central African States to over 90% in the East African Community [29]. Furthermore, the supply of blood from voluntary unpaid donors is insufficient to meet the demand in many countries in SSA. On the other hand, the polarised conceptualisation of voluntary unpaid blood donors and replacement blood donors represents a significant obstacle to constructive discussion surrounding potential innovations to the replacement blood donor systems, which could enhance the blood supply and safety [30].

The implementation of school programmes and community engagement events has led to an improvement in this area in countries such as Malawi. These initiatives have demonstrated efficacy in promoting blood donation and the retention of regular donors [13]. However, it is notable that a relatively low proportion of these voluntary donors remain engaged in the programme over time. Mremi et al. [17] have demonstrated that the majority of these donors are first‐time donors, which presents a similar challenge to that of replacement donors in terms of the prevalence of TTI [17, 23, 24].

The safety of the blood supply is contingent upon suitable donor selection, secure storage assurance, effective processing techniques implementation and donation monitoring [6, 23]. In order to achieve this, potential donors are selected based on whether they exhibit behaviours associated with an increased risk of infection with a transmissible agent. This is done to ensure that donations are made safely and appropriately. In the context of an unidentified risk, the donor’s blood samples are subjected to pathogen and antibody testing to ascertain their suitability for transfusion [6]. These fundamental tenets of transfusion safety are undermined in developing countries like those in the SSA, due to the high endemicity of TTI that characterises them. Furthermore, the selection of donors is frequently inadequate due to their irregularity and the complexity of recruiting voluntary donors [16, 18]. It can be seen that socio‐cultural constraints linked to the influence of a range of beliefs and traditions represent a notable aspect in this context.

Notwithstanding the existing challenges, there are avenues for enhancing the availability of safer blood in the SSA region. This may be achieved primarily through an effective blood donor education and information programme, which would address misinformation and misconceptions about blood donation, as well as insufficient knowledge and awareness of the need for blood donation [30]. Therefore, any intervention must begin with local and culturally appropriate initiatives designed to encourage blood donation [13]. In this context, several initiatives have been initiated in various African countries. For example, peer recruitment programmes in Zimbabwe and South Africa have demonstrated encouraging outcomes concerning the promotion of blood donation. Other interventions that have been proposed or implemented include the use of motivational incentives, reminders and media communications [13]. In this regard, the impact of mobile telephones on this issue is being closely examined in certain countries in southern and western Africa. In both Malawi and the Ivory Coast, there has been a positive response from blood donors to the use of text messages as a reminder system for donations [13]. Moreover, the use of mobile applications in South Africa that facilitate the donation process, provide users with detailed information about their donations, send notifications about upcoming donations, alert users about the status of blood reserves, and offer access to their donation history has been demonstrated to be an effective strategy [13, 31]. The potential for communication through mobile telephones or even social media could be an effective method of promoting blood donation among young people in SSA, who represent the majority of the population. Additionally, they could prove to be an efficient method of transforming non‐blood donors or replacement donors into regular donors [13, 31]. Conversely, as blood donation is primarily contingent upon the general population’s willingness, the involvement of all actors engaged in population mobilisation and education is indispensable. This encompasses the commitment of social leaders, political authorities, blood safety ambassadors and civil society [4].

Encouraging voluntary blood donors will facilitate improvements in transfusion safety. This entails optimising and implementing rigorous donor selection criteria and knowledge dissemination regarding the transmission and prevention of TTI [8, 12, 23]. The initial step in ensuring compliance with the selection criteria is the refinement of the questionnaires designed for donor assessment, adapted to local conditions, which must undergo periodic revision and which is in conjunction with screening tests and related procedures applied to all donations [6, 32, 33]. Therefore, each country should identify the local TTI risk factors and develop an appropriate questionnaire tailored to the specific context [4]. To be most effective, this questionnaire must be administered in an environment of trust and transparency. This will ensure that donors are fully aware of the purpose of the questionnaire and are therefore more likely to provide accurate and truthful responses [34]. In addition to the risk factors for the main TTI—namely sexual behaviour, risky activities and medical interventions—it is essential to consider the local infectious realities and the possibility of travel to endemic areas for certain germs, as outlined in the questionnaire [32]. Blood bank personnel must ensure a comprehensive understanding of the questionnaire, including any potentially sensitive questions. This is to guarantee the veracity and sincerity of the responses provided. This can be achieved by surmounting the challenges associated with multilingualism, which are prevalent in many African countries.

## 4. Blood Donation Screening Tests

To enhance transfusion safety, it is necessary to implement a systematic screening process for all donations for TTI. This process should be carried out in conjunction with the approved kits and the utilisation of confirmatory strategies, consistently quality‐controlled [4, 7, 35]. The licensed assays, which include enzyme‐linked immunosorbent assays and chemiluminescence immunoassays for the detection of antibodies and/or antigens, are suitable for high‐throughput output testing; however, they require a range of specific equipment that currently constitutes the mainstay of blood screening in Africa, and this may limit their application in certain SSA contexts [4, 14]. One potential solution to this issue would be to centralise the production of blood products, thereby reducing fixed costs and improving product quality. Nevertheless, this would necessitate the resolution of the isolation issue prevalent in numerous nations, which could be a rationale for the hospital and, consequently, local production that currently seems to be a standard practice.

Due to their low cost, lack of necessity for specific equipment or power supply and relative ease of use, rapid diagnostic tests (RDTs) are widely employed for donor screening in the SSA countries [15, 16]. However, these have not been validated in the context of the African blood donor population and are known to exhibit low sensitivity and specificity for the main TTI in comparison to enzyme immunosorbent assay and nucleic acid testing (NAT), as shown in Table 2 [15, 16, 35, 36]. Nevertheless, the development of cost‐effective screening strategies, based on validated RDT algorithms, should be adapted to local operational conditions in order to ensure optimal performance. The use of RDT enables a reduction in the cost of blood collection. Nevertheless, subsequent testing is essential to prevent the occurrence of false negatives and false positives, which could result in the misdiagnosis of healthy donors and an increase in the residual risk of blood transfusion [4, 35].

**Table 2:** Recommendations on the performance of different tools used for the screening of transfusion‐transmitted infections [39–43].

Technics	Infection	Sensitivity	Specificity	WP (days)
RDT	HIV	≥ 99%	≥ 98%	20
	HBV (HBsAg)	100%	≥ 98%	45
	HCV	≥ 98%	≥ 97%	65

IA (CLIA or ELISA)	HIV	100%	≥ 98%	19
	HBV (HBsAg)	100%	≥ 98%	30
	HCV	100%	≥ 98%	35

NAT	HIV	> 99%	> 99%	2.93
	HBV			10.34
	HCV			1.34

*Note:* CLIA: chemiluminescence immunoassays; ELISA: enzyme‐linked immunosorbent assays; IA: immunoassays.

Abbreviations: NAT, nucleic acid testing; RDT, rapid diagnostic Test; WP, window period.

A significant challenge faced by low‐income countries is the prevalence of blood‐borne infectious diseases, coupled with the lack of financial resources to implement expensive diagnostic procedures such as viral NAT or pathogen inactivation technology [4, 35]. Such technologies can greatly reduce the window period—the period when donor‐screening markers are not yet detectable but a transfusion is still infectious—for TTI detection. They protect against pathogens, including those with confirmed transfusion‐related risks, such as malaria, thereby diminishing the likelihood of infection [4, 35, 36]. Nevertheless, despite the potential of these technologies to enhance regional blood safety, their deployment faces significant financial barriers, including the cost of reagents and consumables, the need for suitable facilities, the use of sensitive equipment, and the requirement for qualified staff [4, 35, 36]. It is also important to emphasise the lack of effective quality control measures, both in the constitution of stocks and in the use of certain reagents, which contribute to the unreliability of the screening test results [37].

Given the difficulties encountered when seeking to guarantee the quantitative sustainability of the blood supply, it seems reasonable to suggest that a proportion of the available resources should be allocated to obtain precise results concerning certain TTIs. This approach would facilitate the exclusion of unsuitable donors, thereby reducing both the unnecessary deferral of healthy donors and associated costs [33, 35, 38]. This necessitates a considerable increase in the application of specific confirmatory algorithms that take into account local epidemiological conditions and available resources, thus minimising the incidence of false‐positive outcomes [35, 38]. In addition to the aforementioned recommendations for laboratory testing procedures, implementing an effective quality system that encompasses organisational management, quality standards, traceability, equipment qualification, reagent verification before use, regular staff training and participation in proficiency studies has the potential to reduce false positive outcomes further [4, 35].

## 5. Quality Assurance Procedures in the Laboratory Setting

The implementation of quality assurance systems was identified as being of critical importance for the safety of blood and transfusion procedures [4, 37, 44]. In numerous resource‐limited countries, an insufficient blood supply is frequently compounded by inadequate funding, insufficient government legislation and regulation [37]. In addition to the necessity of periodically reassessing the criteria for selecting donors under the evolving epidemiology of diseases and the validation of the questionnaire, the quality system in the transfusion chain continues to present significant challenges and is still found to be limited in SSA blood service data [4, 29]. Indeed, the 2020 WHO African survey revealed that less than 60.0% of SSA countries participated in a national external quality assessment study for TTI and laboratory screening [29]. The efficacy of the screening tests is frequently undermined by many factors, including low reliability, inadequate methodology, inappropriate or unsupported applications and the inability to detect infections in their window period. Furthermore, evidence of noncompliance with the established biosecurity protocols has also been documented [7, 25]. Additionally, it is important to highlight the lack of confirmatory tests for TTI, which has resulted in a significant rate of blood rejection due to the high prevalence of antibodies among donors [33, 35, 38].

To address these issues, a quality system standard tailored specifically for the SSA has been developed since 2014 by the AfSBT. This standard has been designed to improve the quality of both services and blood products [4]. The implementation of the standards through a training and accreditation programme, aligned with the anticipated standards for a given resource level, has proven effective across numerous countries. Nevertheless, it is essential to evaluate the extent to which this approach can foster a sustainable, positive impact on blood safety [4, 45].

## 6. Haemovigilance System Implementation

Adverse events may occur at any stage of the blood product lifecycle, from collection to transfusion to the patient, and may affect donors, recipients and the products themselves [46, 47]. Although haemovigilance plays an essential role in ensuring the quality and safety of blood transfusions, numerous countries lack effective national programmes in this regard [48]. Specific challenges are encountered depending on the organisation of the blood transfusion system in a country [48]. Indeed, the WHO survey on improving national haemovigilance programmes revealed that areas requiring priority action included the availability of sufficient financial resources, the need for personnel training, and the development of guidance documents and data entry systems, as well as the creation of a reliable traceability system or its improvement where it exists [47].

A national haemovigilance system is based on a coordinated approach to continually enhancing the safety, accessibility and suitable utilisation of blood and blood products and related operations across all institutions involved in the transfusion process [46]. This includes the creation of an optimal structure for a national haemovigilance system, its organisational framework, and the identification of relevant entities that will be subject to accountability. These measures are intended to create an environment characterised by the presence of a regulatory programme for the safe production and distribution of blood and blood components [4, 46, 47]. The implementation of national haemovigilance systems remains highly variable. The majority of countries in Africa have operational national blood transfusion programmes. These programmes predominantly emphasise techniques designed to facilitate an increase in regular blood donations, which is intended to enhance the safety of the blood supply. This is accomplished through the establishment of a national blood policy, the provision of staff training and the development of a haemovigilance system [13, 17, 18].

The effectiveness and efficiency of any haemovigilance system are contingent upon a number of key factors. The most important elements are bidirectional traceability—the link from donor to recipient and vice versa—feedback loops, preventive actions and promotion, in addition to confidentiality and the ability to act independently [46, 47]. Furthermore, it is imperative to ensure collaboration with all relevant professional groups in order to facilitate the translation of lessons learned into the prevention of harm and improvement of quality, safety and outcomes [48].

## 7. Human and Operational Resources

Securing the blood supply in some African countries is a significant challenge. These deficiencies can be attributed to several factors, including shortcomings in blood policy, regulatory procedures, financial resources, collection processes, testing methodologies and post‐transfusion monitoring. There is also an acute shortage of qualified and competent support staff with the required skills and expertise to guarantee the reliability of the transfusion chain [4, 49, 50]. The findings of a study conducted at various medical facilities in Cameroon that perform blood transfusions indicate a notable deficit in the knowledge base of staff concerning the identification of suitable donors. Only a third of respondents were aware of the essential tests that must be conducted on a donor’s blood before transfusion [49]. In light of these observations and those in other countries like Ghana, it is recommended that all personnel involved in blood transfusion undergo comprehensive training on the selection and evaluation of donors [49, 51].

The safety of TTI can be enhanced by adopting three key strategies: more rigorous medical selection of blood donors, improved detection of TTI, and advanced physical and chemical treatment of blood components [4]. This should be accompanied by a robust regulatory framework development for in vitro diagnostic medical devices, which is currently underdeveloped. Such a framework is essential to mitigate the risk of finding medical devices with lower quality or lower sensitivity on the market, which could significantly increase the transmission of TTI [15]. Finally, the dearth of qualified human resources, combined with the difficulties presented by suboptimal supply chains, the elevated cost of reagents, the vulnerability of cold chain management, and the unreliability of electrical supply, collectively serve to reinforce the necessity for the utilisation of RDT [15, 52, 53].

## 8. Financial Resources

The financing of the African health system, in general, and the transfusion chain, in particular, is subject to the prevailing economic conditions in the region. Maintaining the availability of blood for transfusion is one of the major expenses for health services [4]. Moreover, the allocation of funding is contingent on a range of considerations, including assessments of blood safety, health economics, budgetary impact and cost‐effectiveness, operational risk and ethical and social concerns [54]. A lack of resources limits the capacity of many blood transfusion services to screen for TTI adequately and safely [13]. Additionally, when action must be taken regarding a disease, it is essential to consider its severity, its epidemiological characteristics, the criteria used for donor selection and the exclusion of high‐risk donations, the characteristics of the test employed and the cost‐effectiveness ratio [6].

It is nonetheless important to underscore that international funding from organisations such as the WHO or other countries plays a crucial role in enhancing blood safety in these countries. This is achieved through an increase in the recruitment of voluntary donors and an expansion in the screening of primary TTI [15]. One of the limitations of this financing system is that it often comes with restrictions requiring strategy implementation that is not always based on local SSA data but rather on the experience of countries that support them [4, 13]. To circumvent these shortcomings, local governments must identify alternative approaches, such as prioritising the establishment of robust financing systems for blood transfusion services with a focus on public‐private partnerships [1, 13]. Moreover, a financially sustainable model could include a cost‐recovery strategy, such as the distribution of the cost of blood products across various sectors and stakeholders, including hospitals, governments, health insurance providers and patients. Another option could be the engagement of the community to support transfusion services and ensure the security of the blood supply [4, 36].

## 9. Prospects

The safety of blood and blood components has been largely contingent on two primary strategies: first, the exclusion of donors who may be at heightened risk of infection based on epidemiological profiles of disease; and second, the development and deployment of screening tests designed to identify and exclude donations that may be infected [55]. Nonetheless, there are some infections for which no suitable tests are available. Consequently, the risk reduction is contingent upon the potential donor’s exclusion based on screening questions or physical approaches, such as pathogen reduction technologies [6, 25, 32, 56]. These technologies are specifically designed to target nucleic acids, thereby rendering known emerging or re‐emerging viruses, bacteria and protozoa in blood products inactivated. These technologies could provide a potential benefit for known and unknown infections and thus represent a final palliative when traditional screening methods fail to detect the responsible pathogens [6, 25, 56]. Nevertheless, if the widespread introduction of these technologies is to move forward in Africa, operational research is needed to assess how these technologies can be integrated practically, given the extant challenges of low‐resource settings. Thus, the possibility of several countries combining to support a multinational or regional testing facility also needs consideration [4].

The challenge of acquiring sufficient quantities of blood of the requisite quality in the SSA underscores the necessity for more rigorous research to establish more effective health policies for transfusion safety [1, 8]. There is an urgent need to design and implement innovative communication approaches to strengthen blood transfusion services through social media and mass media interventions [13, 31]. Similarly, initiatives should be implemented to transform replacement donors or even non‐donors into regular blood donors. Moreover, given the close relationship between national transfusion services and governmental agencies, the establishment of sustainable financial strategies through local partnerships can facilitate not only the creation of a stable blood supply but also the enhancement of quality and safety standards throughout the entire blood supply chain. This includes the recruitment of donors, the distribution of blood and the processes of blood collection, analysis and treatment [13, 36]. Ultimately, beyond the control of pre‐analytical conditions and screening tests, introducing NAT could reduce the incidence of infection transmission by transfusion. However, this would necessitate a satisfactory and cost‐effective reporting system implementation [22, 33, 52].

There is evidence of a decline in the incidence of TTI, such as HBV, among the donor population. Indeed, a South African study demonstrated a reduction in the risk of HBV transfusion transmission as a greater proportion of vaccinated young people joined the donor pool and became regular donors, thus improving the security of the supply [57]. This may be attributed to the efficacy of the HBV vaccination in preventing infection in individuals who have received the full course of vaccination [7, 33]. It is therefore imperative to maintain the ongoing efforts to achieve complete vaccination coverage in different African countries. The implementation of a full vaccination programme against HBV has the potential to reduce up to half the TTI risks. However, the cost of such a programme must be included in a sustainable funding approach for the blood services [4, 29]. Similarly, the gradual incorporation of the malaria vaccine into national immunisation programmes represents a more optimal strategy for malaria prevention, given the complexity of alternative methods, as previously discussed.

Government involvement in the blood transfusion chain should include the development and implementation of appropriate blood regulations and policies, as well as appropriate governance, financial support and flexible collaboration in solving ongoing technical and financial issues [4]. Nevertheless, implementing an effective haemovigilance system is a fundamental part of a comprehensive structural framework designed to minimise the incidence of TTI in patients within this specific geographical region [7, 47, 50]. Despite the relatively limited role of haemovigilance in the detection of new blood‐borne infectious diseases, the continuous monitoring of transfusion‐related infections allows for the observation of patterns in the transmission of infectious diseases and the evaluation of the impact of novel pathogenic agents within the transfusion chain [48]. Moreover, the integration of serosurveillance and haemovigilance with infectious disease case surveillance represents an effective strategy for optimising the capacity of the entire supply chain logistics to respond safely to demand [58]. Haemovigilance systems that are well established may be relied upon to provide data of an acceptable quality that may be of use in developing, evaluating and improving transfusion systems in contexts of limited resources [48]. Finally, it is essential to adapt the configuration and scope of haemovigilance systems to align with the specific needs of individual establishments and countries. In countries where the haemovigilance programme is still under development, it can be beneficial to concentrate on the measures that can be implemented gradually to achieve a more comprehensive programme [46, 47].

At last, natural disasters and pandemics can be highly challenging to blood supply chains [58]. In recent years, two significant health crises have emerged as public health emergencies of international concern. The first health crisis, the coronavirus disease 2019 (COVID‐19), and the consequent lockdown have resulted in a progressive decline in blood donations [59–61]. Regarding the monkeypox virus (MPXV) epidemic, its epidemiology and its systemic spread and viraemia during acute infection represent a potential threat to blood transfusion safety [62]. Similar to the Ebola virus, the Marburg virus (MARV) is a highly contagious pathogen that causes severe viral haemorrhagic fever in humans [63, 64]. There is evidence that this filovirus has been transmitted outside of known endemic areas and thus remains undetected by current surveillance efforts [65]. Indeed, the characteristics of MARV disease survivors and asymptomatic viraemia remain poorly understood. While transmission via transfusion has not been documented, instances of transmission via contact with blood have been reported in clinical cases [64]. In summary, the emergence of COVID‐19, MPXV and MARV demonstrates the potential impact of novel infectious diseases on the safety and adequacy of the blood supply [64]. To effectively address future crises, blood transfusion services may consider numerous strategies. These strategies must include health worker training, technological innovations, information monitoring improvement, and direct evaluation of transmission risk until appropriate techniques have been validated [66].

## 10. Conclusion

The use of blood and blood products carries an inherent risk of morbidity and mortality. TTIs can greatly affect the health and well‐being of transfusion recipients in SSA countries. SSA countries are confronted with a plethora of challenges in their attempts to regulate TTI. These challenges include the inadequacy of TTI screening procedures, the absence of a robust quality system and the dearth of effective control mechanisms. Nevertheless, the aforementioned countries are currently engaged in a range of efforts to confront these challenges. Furthermore, they have also developed a variety of solutions that have been adapted to suit local contexts. These innovative solutions are already being implemented in several SSA countries.

## Disclosure

All contributors have been listed as co‐authors with their permission.

## Conflicts of Interest

The authors declare no conflicts of interest.

## Author Contributions

Each author played a significant role throughout every stage of the research.

F. Y. Djientcheu Deugoue: conceptualisation, methodology, resources, investigation, writing–original draft, writing–review and editing, validation.

M. Azabji‐Kenfack: software, resources, review and editing, validation.

C. Tayou Tagny: resources, writing–review and editing, validation.

M. A. Kouamou Tchiekou: writing–original draft, writing–review and editing.

S. A. Menteng Tchuente: writing–original draft, writing–review and editing.

Veronique Deneys: conceptualisation, writing–review and editing, supervision, validation.

## Funding

No funding was received for this work.

## Data Availability

Data sharing is not applicable to this article as no datasets were generated or analysed during the current study.
